# Glucose and fatty acid metabolism involved in the protective effect of metformin against ulipristal-induced endometrial changes in rats

**DOI:** 10.1038/s41598-021-88346-w

**Published:** 2021-04-23

**Authors:** Marwa S. Hamza, Eman Ramadan, Salama A. Salama

**Affiliations:** 1 Clinical Pharmacy Practice Department, Faculty of Pharmacy, The British University in Egypt, El-Sherouk City, P.O. Box 43, Cairo, 11837 Egypt; 2 The Center for Drug Research and Development (CDRD), Faculty of Pharmacy, The British University in Egypt, El-Sherouk City, P.O. Box 43, Cairo, 11837 Egypt; 3 Pharmacology and Biochemistry Department, Faculty of Pharmacy, The British University in Egypt, El-Sherouk City, P.O. Box 43, Cairo, 11837 Egypt; 4 Pharmacology and Toxicology Department, Faculty of Pharmacy, Al-Azhar University Nasr City, Cairo, 11751 Egypt

**Keywords:** Molecular biology, Systems biology, Gynaecological cancer

## Abstract

Ulipristal acetate (UPA) is effective in the treatment of uterine fibroids. However, its clinical use is hampered by the development of pathologic progesterone receptor modulator-associated endometrial changes (PAECs). The current study was designed to test the hypothesis that UPA-induced PAECs are associated with deranged expression of some metabolic genes. In addition, metformin can mitigate UPA-induced PAECs through modulating the expression of these genes. In the present study, twenty-eight female non-pregnant, nulligravid Wistar rats were treated with UPA (0.1 mg/kg/day, intragastric) and/or metformin (50 mg/kg/day, intragastric) for 8 weeks. Our results demonstrated that co-treatment with metformin significantly reduced UPA-induced PAECs. In addition, co-treatment with metformin and UPA was associated with significant increase in the Bax and significant reduction in Bcl-2, PCNA, Cyclin-D1and ER-α as compared to treatment with UPA alone. Furthermore, treatment with UPA alone was associated with deranged expression of 3-phosphoglycerate dehydrogenase (3-PHGDH), glucose-6-phosphate dehydrogenase (G6PD), transketolase (TKT), fatty acid synthase (FAS) and CD36. Most importantly, co-treatment with metformin markedly reduced UPA-induced altered expression of these metabolic genes in endometrial tissues. In conclusion, UPA-induced PAECs are associated with altered expression of genes involved in cell proliferation, apoptosis, estrogen receptor, glucose metabolism and lipid metabolism. Co-treatment with metformin abrogated UPA-induced PAECs most likely through the modulation of the expression of these genes.

## Introduction

Ulipristal acetate (UPA) is a selective progesterone receptor modulator (SPRM). It has tissue selective, mixed progesterone agonist and antagonist effects in myometrial and endometrial tissues^1,2^. By the virtue of its SPRM activity, UPA offers a unique potential for clinical application in gynecology^3^. It is used as a single dose contraceptive to suppress or delay ovulation^4,5^. In addition, in women with symptomatic fibroids it reduces fibroid size due to its antiproliferative, antifibrotic and pro-apoptotic effects on the fibroid^6,7^.

The extended use of UPA is associated with non-physiological histopathological endometrial changes, collectively known as progesterone receptor modulator-associated endometrial changes (PAECs)^3^. PAECs have similar appearance to tamoxifen-induced endometrial changes and are associated with endometrial thickening^8^. At the histological level, PAECs are characterized by a transient increase of endometrial thickness, cystically dilated glands, epithelial distortion, apoptosis and low mitotic activities in glands and stroma^8^. In addition, the unexplained proliferation of the endometrial epithelium leads to changes in the size and shape of the endometrial glands and increase in the ratio of endometrial glands and stroma leading to endometrial hyperplasia^9,10^. Substantial evidence indicates that PAECs rapidly regress on cessation of treatment, although the rate of regression can be variable^11^. Whilst PAECs are now well described and appear reversible, the mechanisms by which they develop are poorly understood^12^.

Growing evidence suggest that the development and progression of some premalignant lesions is associated with metabolic reprogramming in the normal-appearing precancerous lesions^13^. Additionally, emerging evidence highlighted the important role of metabolic reprogramming of the microenvironment and macroenvironment in the development of pathophysiological changes in the endometrium^14,15^. Indeed, previous work from our group and others demonstrated that sex hormones and their cognate receptors regulate endometrial metabolism^16,17^.

Clinical and pre-clinical studies demonstrated that metformin, the cornerstone oral agent for the treatment of type 2 diabetes, plays a role in modulating cell metabolism and therefor it inhibits the development and progression of cancerous and precancerous lesions. Metformin may have a beneficial therapeutic effect in endometrial cancer^18^, due to its indirect effects within the metabolic milieu and direct effect on endometrial cells through AMPK activation/mTOR inhibition and suppression of fatty acid/lipid biosynthesis^19^. Metformin decreases proliferation by lowering lipid synthesis used for membrane biosynthesis^20^. Metformin down-regulates the effects of various pro-oncogenic pathways including insulin/PI3K/Akt, and c-Myc signaling^21,22^. In addition, previous studies confirmed the insulin sensitizing effect of metformin on endometrial hyperplasia^23,24^, endometrial carcinoma^25^ and atypical endometrial hyperplasia^26^. This effect is due to reducing endogenous insulin and activating the enzyme adenosine monophosphate-activated protein kinase (AMPK), which can inhibit the synthesis of cell proteins and ultimately cell growth^27^.

An increasing body of evidence suggests that obesity, diabetes and insulin resistance are strong risk factors for endometrial carcinoma. Insulin-like growth factors play a major role in carcinogenesis and cancer progression^28^. Furthermore, it has been shown that insulin has a role throughout the menstrual cycle of healthy women affecting the stromal cell decidualization^29^. The relationship between endometrial hyperplasia, insulin, insulin’s mediators and insulin’s sensitizers^30^ raises the prospect of targeting these metabolic changes as a viable target for preventing or treating these conditions. However, it remains largely unknown whether UPA rewires endogenous metabolic programs in endometrial tissues and whether this contributes to UPA-induced histopathological changes in the endometrium. Therefore, in this study, we assessed whether metformin can circumvent UPA-induced pathological changes in rats endometrium through its pleiotropic effects of cellular metabolism.

## Results

### Effect of ulipristal or/and metformin on rats’ uterine weight

**Figure 1:**
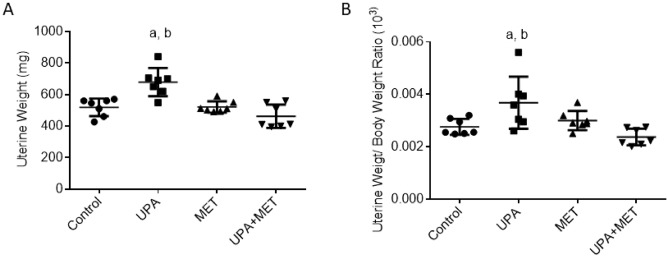
Effect of ulipristal or/ and metformin treatment on the rats’ uteri weight (**A**), uterus weight/body weight (**B**). Data is represented by mean ± SD (n = 7). a or b, Statistically significant from control or ulipristal and metformin treated group respectively at *P* < 0.05 using one-way ANOVA followed by Tukey as a post hoc test. *UPA* ulipristal, *MET* metformin, *UPA + MET* co-treatment with ulipristal and metformin.

Based on the well-established fact that progesterone affects the uterine weight, we began with the assessment of the effect of UPA or/and metformin treatment on the uterine weight. The UPA-treated group showed a significant increase in the average weight of uteri and the uteri weight/body weight ratio by 30% and 33% respectively as compared with the normal control group (*P* < 0.05, Fig. 1). Significantly, co-treatment with metformin markedly reduced the average weight of uteri and the uteri weight/body weight ratio by 32% and 35% as compared to animals treated with UPA alone (*P* < 0.05, Fig. 1).

### Effect of ulipristal or/and metformin in histopathology of rats’ endometrium

**Figure 2:**
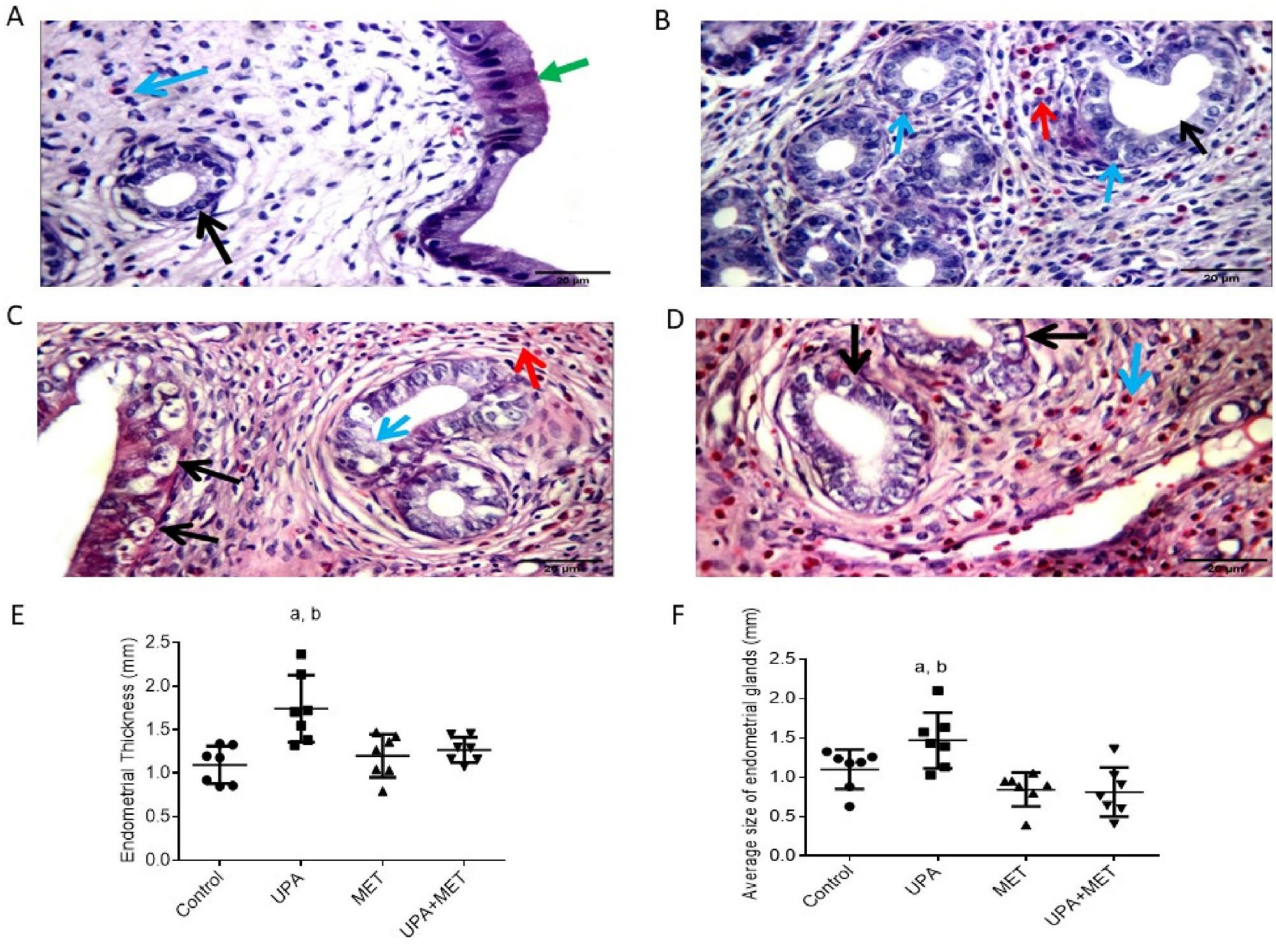
Representative images from each group of rats to demonstrate the histological examination of hematoxylin–eosin sections of rat uteri and the effect of ulipristal or/and metformin treatment on the rats’ endometrial thickness, glandular area. H&E × 400 magnification (scale bar = 20 μm). (**A**) Section taken from the uteri of the control group, the uterine wall showing average surface epithelium (green arrow) with uniform rounded endometrial gland (black arrow) in cellular stroma (blue arrow). (**B**) Section taken from the uteri wall of rats treated with UPA alone. It showed angulated and rounded glands (black arrow) with nuclear pseudo stratification (blue arrows) in compact cellular stroma showing excess eosinophils (red arrow). (**C**) Section taken from the uteri from metformin-treated rats, uterine wall showing variable-sized endometrial glands with sub nuclear (black arrows) and supra-nuclear secretory vacuoles (blue arrow) with excess stromal eosinophils (red arrow). (**D**) Section taken from rats co-treated with metformin and UPA, uterine wall showing uniform rounded endometrial glands lined by vacuolated cells (black arrows) in cellular stroma with excess eosinophils (blue arrow). (**E**) Effect of ulipristal or/and metformin treatment on the rats’ endometrial thickness. (**F**) Effect of ulipristal or/and metformin treatment on the rats’ glandular area. Data in figures (**E**) and (**F**) is represented by mean ± SD (n = 7). a or b, Statistically significant from the control or ulipristal & metformin treated group respectively at *P* < 0.05 using one-way ANOVA followed by Tukey as a post hoc test. *UPA* ulipristal, *MET* metformin, *UPA + MET* co-treatment with ulipristal and metformin.

Then, we assessed the effects of UPA or/and metformin treatment on uterine histologic parameters. In the uteri from the control group or the group treated with metformin alone, the endometrium showed normal histological features. Thus, analysis of the uteri from the control group showed that the luminal epithelial cells remained cuboidal with similar thickness and showed normal glandular/stromal ratio with uniform rounded equally distributed endometrial gland lined by single-cell layer with basally located nuclei in cellular stroma showing excess eosinophils. (Fig. 2A). Similarly, in the uteri from metformin-treated rats, the uterine wall showed average glandular/stromal ratio with equally distributed rounded endometrial glands with sub nuclear and supra-nuclear secretory vacuoles with excess stromal eosinophils, edematous stroma with average myometrium (Fig. 2C). In contrast, the uteri wall of rats treated with UPA alone showed hyperplasic changes, increased glandular/stromal ratio, variable-sized cystically dilated glands, compact cellular stroma with surface stromal edema, under developed spiral arterioles, excess eosinophils and average myometrium with congested blood vessels (Fig. 2B). In rats co-treated with metformin and UPA, the uteri wall showed average glandular/stromal ratio with equally distributed rounded endometrial glands, edematous stroma (Fig. 2D).

### Effect of ulipristal or/and metformin on rats endometrial thickness

The thickness of endometrium, the vertical distance from the junction of endometrium and myometrium to uterine cavity, were determined in rats from different treatment groups. In the normal control group or the group treated with metformin alone, the average endometrial thicknesses were 1.09 mm and 1.2 mm, respectively. While in rats treated with UPA alone, the endometrial thickness was 1.7 mm which represents an increase by 70% when compared with normal control group (*P* < 0.01). In the group co-treated with metformin and UPA, the endometrial thickness was 1.3 mm which represents a decrease by 25% when compared with rats treated with UPA alone (*P* < 0.01) (Fig. 2E).

Regarding the glandular area, in the normal control group and the group treated with metformin alone, the average glandular areas were 1.1 mm and 0.8 mm, respectively. While in rats treated with UPA alone, the glandular area was 1.5 mm represents an increase by 33% when compared with normal control group (*P* < 0.05). In the group co-treated with metformin and UPA, the glandular area was 0.8 mm which represents a decrease by 45% when compared with rats treated with UPA alone (*P* < 0.05) (Fig. 2F).

### Effect of metformin or/and ulipristal on the expression of apoptosis in rats’ endometrium

**Figure 3:**
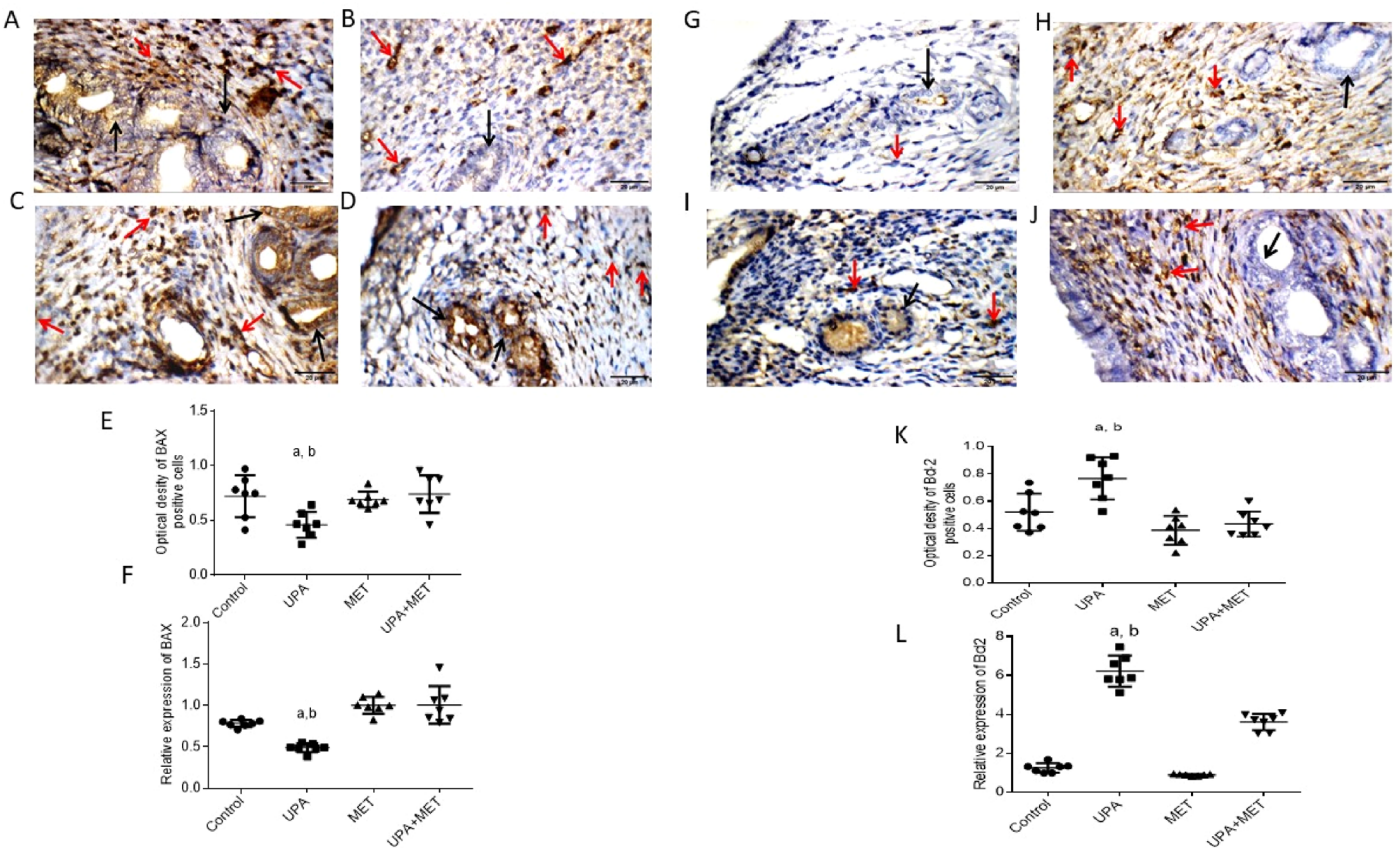
Effect of metformin or/and ulipristal on the expression of the apoptosis markers of rats’ uteri. (**A**–**D**) photomicrographs of uteri shows expression of apoptotic marker Bax. (**A**) Uterine wall in the control group. (**B**) Uterine wall in ulipristal treated group. (**C**) Uterine wall in metformin treated group. (**D**) Uterine wall in the ulipristal and metformin treated group. × 400 magnification (scale bar = 20 μm). (**E**) Expression quantification of the optical density reactivity of positive cells of Bax by the ImageJ analysis system. (**F**) Quantitative RT-PCR analysis of Bax mRNA expression. (**G**–**J**) photomicrographs of uteri shows expression of anti-apoptotic marker Bcl-2. (**G**) Uterine wall in the control group. (**H**) Uterine wall in ulipristal treated group. (**I**) Uterine wall in the metformin treated group. (**J**) Uterine wall in the ulipristal and metformin treated group. × 400 magnification (scale bar = 20 μm). (**K**) Expression quantification of the optical density reactivity of positive cells of Bcl-2 by the ImageJ analysis system. (**L**) Quantitative RT-PCR analysis of Bcl-2 mRNA expression. Black arrow refers to reactivity in endometrial glands, red arrows refers to reactivity in stromal cells. Data in figures (**E**) and (**K**) is represented by mean ± SD (n = 7). All value of RT-PCR is expressed as the change in cycle threshold (ΔCt). Each dot represents mean of each group (n = 7, triplicate for each rat). Negative control (DEPC-treated water) showed no detectable fluorescent signals. a or b, Statistically significant from the control or ulipristal and metformin treated group respectively at *P* < 0.05 using one-way ANOVA followed by Tukey as a post hoc test. *UPA* ulipristal, *MET* metformin, *UPA + MET* co-treatment with ulipristal and metformin.

Then, we studied the effect of UPA or/and metformin on the expression of apoptosis and antiapoptotic genes in endometrial tissues at the protein level by IHC staining and the mRNA level by RT-PCR. As shown in (Fig. 3A–D), uterine wall in the control group showed negative reactivity towards BAX in endometrial glands, and moderate cytoplasmic reactivity in stromal cells. While uterine wall in the UPA treated group showed negative reactivity towards BAX in endometrial glands and moderate cytoplasmic reactivity in stromal cells. Uterine wall in the metformin treated group showed marked cytoplasmic reactivity towards BAX in endometrial glands, and moderate reactivity in stromal cells while uterine wall in the UPA and metformin treated group showed marked cytoplasmic reactivity in endometrial glands, and weak reactivity in stromal cells.

The immunoreactivity of Bax in the UPA treated group significantly decreased by one half- fold as compared to both the control group and the group treated with metformin alone (0.45 ± 0.13 vs. 0.7 ± 0.25 and 0.7 ± 0.17; respectively (*P* < 0.01). In the group co-treated with metformin and UPA, the Bax immunoreactivity was increased by one- third fold as compared to rats treated with UPA alone (0.71 ± 0.19 vs. 0.45 ± 0.13; respectively) (*P* < 0.01) (Fig. 3E).

With regard to the effect of treatment with UPA or/and metformin on anti-apoptotic gene Bcl-2 expression in the endometrial tissues, uterine wall in the control group showed negative reactivity towards Bcl-2 in endometrial glands, and negative reactivity in stromal cells. Uterine wall in the UPA treated group showed mild reactivity towards Bcl-2 in endometrial glands, and moderate reactivity in stromal cells while uterine wall in the metformin treated group showed negative reactivity in endometrial glands, and weak cytoplasmic reactivity in stromal cells. Uterine wall in the UPA and metformin treated group showed negative reactivity in endometrial glands, and moderate cytoplasmic reactivity in stromal cells) (Fig. 3G–J).

The immunoreactivity of Bcl-2 in the UPA treated group significantly increased by around one third-fold as compared to the control group (0.7 ± 0.19 vs*.* 0.5 ± 0.09) and by half- fold as compared with metformin alone and the group treated with metformin alone (0.7 ± 0.19 vs. 0.4 ± 0.15) respectively) (*P* < 0.01). In the metformin and UPA-treated group, the immunoreactivity of Bcl-2 was decreased by half-fold as compared to rats treated with UPA alone (0.4 ± 0.16 vs. 0.7 ± 0.19; respectively) (*P* < 0.01) (Fig. 3K).

Bax/Bcl-2 ratio was calculated as the percentage of positive Bax divided by the percentage of positive Bcl-2). The Bax/Bcl-2 ratio was > 1 in the UPA-treated group and after adding metformin to UPA the Bax/Bcl-2 ratio become < 1 (*P* < 0.05).

Consistent with the results of IHC, RT-PCR profiling of the mRNA expression of Bax and Bcl-2 revealed that UPA significantly decreased the level of BAX by around one third- fold and one half- fold respectively as compared to both the control group and the group treated with metformin alone (*P* < 0.05). Addition of metformin to UPA significantly increased the level of Bax by onefold as compared to the UPA treated group (*P* < 0.05). UPA significantly increased expression of Bcl-2 by fourfold and sixfold respectively as compared to the control group and the group treated with metformin alone (*P* < 0.05). Addition of metformin to UPA showed significant down-regulation of the levels of Bcl-2 by half- fold as compared to the UPA group (*P* < 0.05). (Fig. 3F, L).

### Effect of ulipristal or/and metformin on rats’ endometrium proliferation

**Figure 4:**
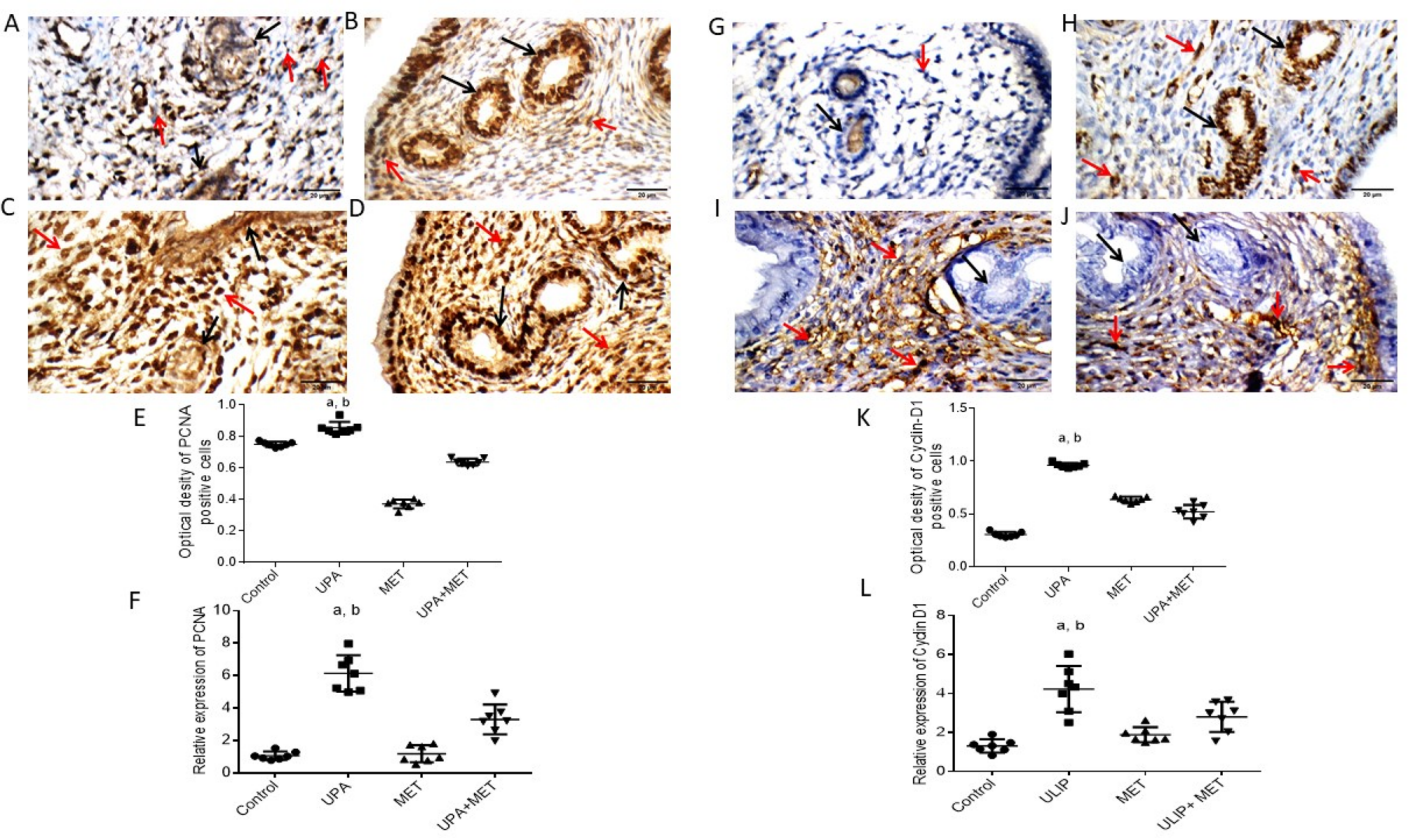
Effect of metformin or/and ulipristal on the expression of the proliferation markers of rats’ uteri. (**A**–**D**) photomicrographs of uteri shows expression of PCNA (**A**) Uterine wall in the control group. (**B**) Uterine wall in ulipristal treated group. (**C**) Uterine wall in metformin treated group. (**D**) Uterine wall in the ulipristal and metformin treated group. × 400 magnification (scale bar = 20 μm). (**E**) Expression quantification of the optical density reactivity of positive cells of PCNA by the ImageJ analysis system. (**F**) Quantitative RT-PCR analysis of PCNA mRNA expression. (**G**–**J**) Photomicrographs of uteri shows expression of Cyclin-D. (**G**) Uterine wall in the control group. (**H**) Uterine wall in ulipristal treated group. (I) Uterine wall in metformin treated group. (**J**) Uterine wall in the ulipristal and metformin treated group. × 400 magnification (scale bar = 20 μm). (**K**) Expression quantification of the optical density reactivity of positive cells by the ImageJ analysis system. (**L**) Quantitative RT-PCR analysis of Cyclin D1 mRNA expression, Black arrow refers to reactivity in endometrial glands, red arrows refers to reactivity in stromal cells. Data in figures (**E**) and (**K**) is represented by mean ± SD (n = 7). All value of RT-PCR is expressed as the change in cycle threshold (ΔCt). Each dot represents mean of each group (n = 7, triplicate for each rat). Negative control (DEPC-treated water) showed no detectable fluorescent signals. a or b, Statistically significant from the control or ulipristal and metformin treated group respectively at *P* < 0.05 using one-way ANOVA followed by Tukey as a post hoc test. *UPA* ulipristal, *MET* metformin, *UPA + MET* co-treatment with ulipristal and metformin.

In order to confirm the effect of metformin on inhibiting UPA-induced endometrial proliferation, IHC staining of the proliferation markers PCNA and cyclin D1 was performed in uterine tissue. Results of immunohistochemistry of PCNA showed that uterine wall in the control group showed weak nuclear reactivity in endometrial glands, and moderate reactivity in stromal cells. While uterine wall in the UPA treated group showed marked nuclear reactivity in endometrial glands, and weak reactivity in stromal cells. Uterine wall in the metformin treated group showed moderate nuclear reactivity in endometrial glands, and marked reactivity in stromal cells. Uterine wall in the UPA and metformin treated group showed marked nuclear reactivity in endometrial glands, and marked reactivity in stromal cells (Fig. 4A–D).

Treatment with UPA significantly increased the immunoreactivity of PCNA by one seventh- fold and one and half-fold as compared to the control group and the group treated with metformin alone (0.8 ± 0.22 vs. 0.7 ± 0.19 and 0.3 ± 0.05; respectively (*P* < 0.05). In the group co-treated with metformin and UPA, the PCNA immunoreactivity decreased by a quarter-fold as compared to UPA group (0.6 ± 0.18 vs. 0.8 ± 0.22) (*P* < 0.05) (Fig. 4E).

Similarly, the IHC results showed that uterine wall in the control group showed negative reactivity towards Cyclin D1 in endometrial glands, and negative reactivity in stromal cells. Uterine wall in the UPA treated group showed marked nuclear reactivity in endometrial glands, and moderate reactivity in stromal cells. Uterine wall in the metformin treated group showed negative reactivity for Cyclin D1 in endometrial glands, and moderate nuclear reactivity in stromal cells. Uterine wall in the UPA and metformin treated group showed negative reactivity in endometrial glands, and moderate nuclear reactivity in stromal cells (Fig. 4G–J). Treatment with UPA significantly increased immunoreactivity of cyclin-D1 by twofold and half-fold as compared to the control group and the group treated with metformin alone (0.9 ± 0.29 vs. 0.3 ± 0.09 and 0.6 ± 0.18; respectively) (*P* < 0.05). In the group co-treated with metformin and UPA, the cyclin-D1 immunoreactivity decreased by around one half- fold as compared to UPA group (0.5 ± 0.1 vs*.* 0.9 ± 0.29) (*P* < 0.05) (Fig. 4K).

The observations detected by IHC staining were confirmed by RT-PCR profiling of the mRNA expression of PCNA and cyclin D1. UPA significantly increased the level of PCNA by about 5-fold as compared to both control group and the group treated with metformin alone (*P* < 0.05). Addition of metformin significantly decreased the expression level of PCNA by half-fold as compared to UPA group (*P* < 0.05). UPA significantly increased expression of cyclin D1 mRNA by twofold and onefold respectively, as compared to the control group and the group treated with metformin alone (*P* < 0.05). Addition of metformin to UPA showed significant down-regulation of the levels of cyclin D1 mRNA by one third- fold as compared to the UPA group (*P* < 0.05) (Fig. 4F, L).

### Effect of ulipristal or/and metformin on ERα and PR expression in rats’ endometrium

**Figure 5:**
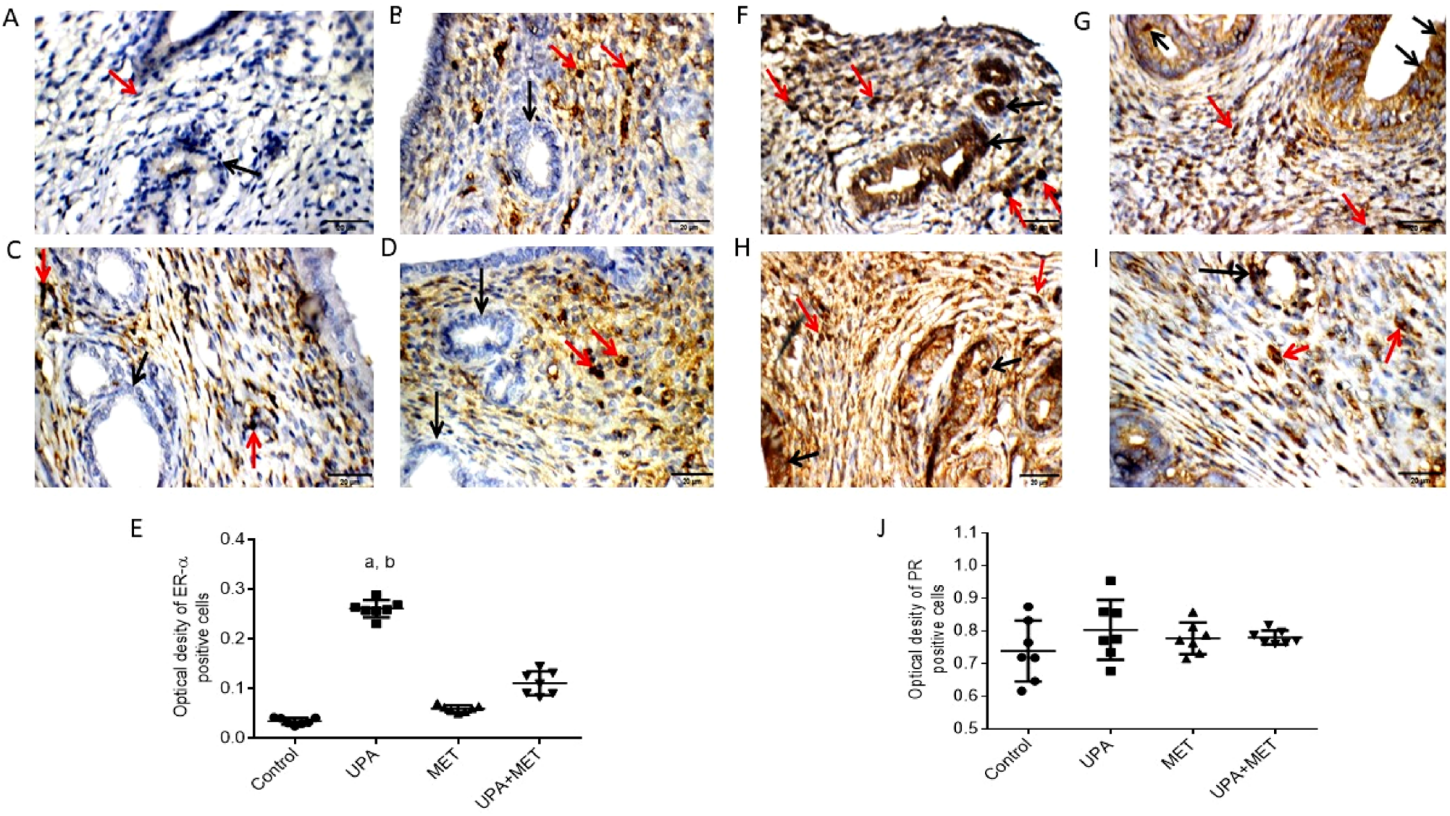
Effect of ulipristal or/and metformin on ERα and PR expression of rats’ uteri. (**A**–**D**) photomicrographs of uteri show expression of ERα. (**A**) Uterine wall in the control group. (**B**) Uterine wall in ulipristal treated group. (**C**) Uterine wall in metformin treated group. (**D**) Uterine wall in the ulipristal and metformin treated group. × 400 magnification (scale bar = 20 μm). (**E**) Expression quantification of the optical density reactivity of positive cells of ERα by the ImageJ analysis system. (**F**–**I**) photomicrographs of uteri shows expression of PR. (**F**) Uterine wall in the control group. (**G**) Uterine wall in ulipristal treated group. (**H**) Uterine wall in metformin treated group. (**I**) Uterine wall in ulipristal and metformin treated group. × 400 magnification (scale bar = 20 μm). (**J**) Expression quantification of the optical density reactivity of positive cells of PR by the ImageJ analysis system. Data in figures (**E**) and (**J**) is represented by mean ± SD (n = 7). a or b, Statistically significant from the control or ulipristal and metformin treated group respectively at *P* < 0.05 using one-way ANOVA followed by Tukey as a post hoc test. *UPA* ulipristal, *MET* metformin, *UPA + MET* co-treatment with ulipristal and metformin.

Then, we investigated the effect of UPA or/and metformin on ERα and PR expression in endometrium by immunohistochemical staining. IHC results showed that uterine wall in the all the groups showed negative reactivity towards ERα in endometrial glands but only the control group showed negative reactivity in the stromal cells. The other 3 groups; UPA treated group, metformin treated group as well as UPA and metformin treated group showed weak nuclear reactivity in stromal cells (Fig. 5A–D). In conclusion, treatment with UPA significantly increased the immunoreactivity of ERα by 7.6-fold and threefold as compared to the control group and the group treated with metformin alone (0.26 ± 0.05 vs*.* 0.03 ± 0.01 and 0.06 ± 0.02; respectively) (*P* < 0.05). In the group co-treated with metformin and UPA, the ERα immunoreactivity decreased by 1.6-fold as compared to UPA group (0.26 ± 0.05 vs*.* 0.1 ± 0.02) (*P* < 0.05) (Fig. 5E).

The IHC results of PR showed that uterine wall in the control group showed marked nuclear reactivity towards PR in endometrial glands and moderate reactivity in stromal cells. Also, uterine wall in the UPA treated group showed weak nuclear reactivity in endometrial glands, and weak reactivity in stromal cells. Uterine wall showed marked nuclear reactivity in endometrial glands, and moderate reactivity in stromal cells. Uterine wall in the UPA and metformin treated group showed weak nuclear reactivity for PR in endometrial glands, and moderate reactivity in stromal cells (Fig. 5F–I). Treatment with UPA insignificantly increased immunoreactivity of PR as compared to the control group and the group treated with metformin alone. Moreover, in the group co-treated with metformin and UPA, the PR immunoreactivity insignificantly decreased when compared to UPA group (Fig. 5J).

### Effect of ulipristal and/or metformin on the expression of proteins involved in cellular metabolism

**Figure 6:**
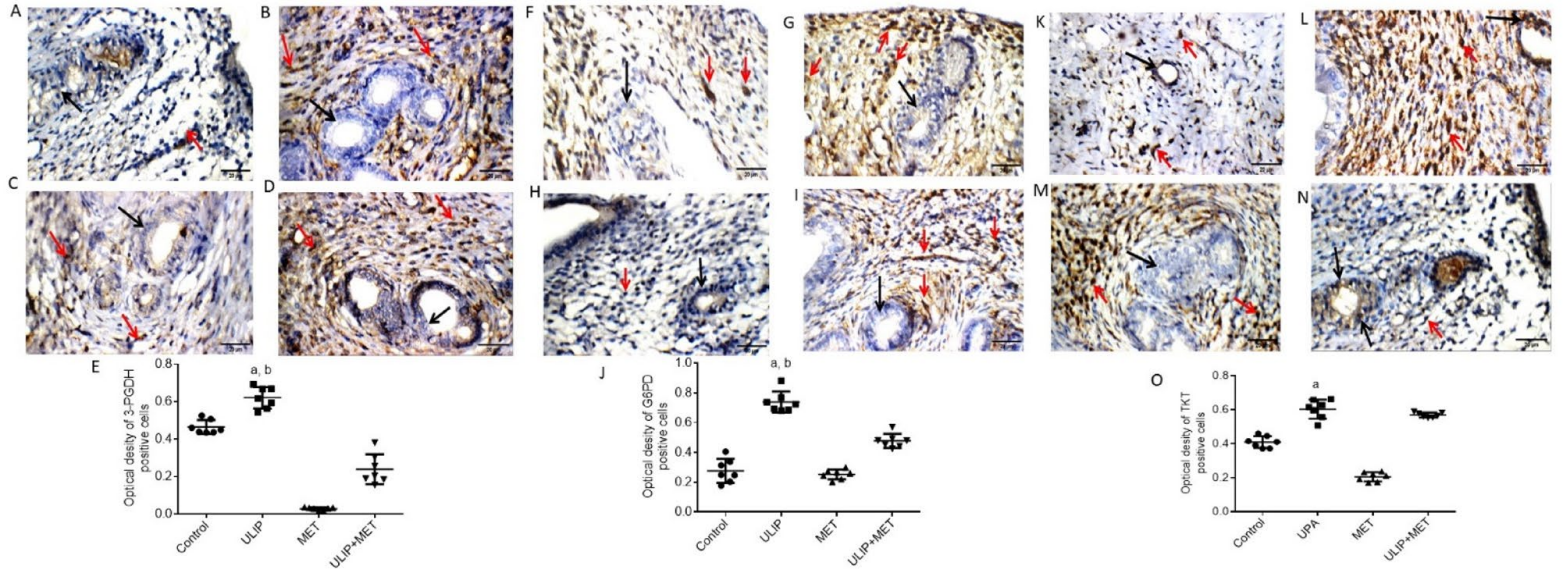
Effect of ulipristal and/or metformin on the expression of proteins involved in glucose metabolism in rats’ uteri. (**A**–**D**) photomicrographs of uteri shows expression of 3-PHGDH. (**A**) Uterine wall in the control group. (**B**) Uterine wall in ulipristal treated group. (**C**) Uterine wall in metformin treated group. (**D**) Uterine wall in the ulipristal and metformin treated group. × 400 magnification (scale bar = 20 μm). (**E**) Expression quantification of the optical density reactivity of positive cells of 3-PHGDH by the ImageJ analysis system. (**F**–**I**). Photomicrographs of uteri shows expression of G6PD. (**F**) Uterine wall in the control group. (**G**) Uterine wall in ulipristal treated group. (**H**) Uterine wall in metformin treated group. (**I**) Uterine wall in the ulipristal and metformin treated group. × 400 magnification (scale bar = 20 μm). (**J**) Expression quantification of the optical density reactivity of positive cells of G6PD by the ImageJ analysis system. (**K**–**N**) Photomicrographs of uteri shows expression of TKT. (**K**) Uterine wall in the control group. (**L**) Uterine wall in ulipristal treated group. (**M**) Uterine wall in metformin treated group. (**N**) Uterine wall in ulipristal and metformin treated group. × 400 magnification (scale bar = 20 μm). (**O**) Expression quantification of the optical density reactivity of positive cells of TKT by the ImageJ analysis system. Data in figures (**E** & **J** & **O**) is represented by mean ± SD (n = 7). a or b, Statistically significant from the control or ulipristal & metformin treated group respectively at *P* < 0.05 using one-way ANOVA followed by Tukey as a post hoc test. *UPA* ulipristal, *MET* metformin, *UPA + MET* co-treatment with ulipristal and metformin.

To determine whether UPA-induced endometrial changes are accompanied by alteration in the endometrial metabolism, we examined the expression of 3-Phosphoglycerate dehydrogenase (3-PHGDH), the first rate-limiting enzyme of serine synthesis which is frequently overexpressed in proliferating tissues. 3-PHGDH overexpression directs glucose metabolism towards serine synthesis to promote cell proliferation^31^. Because reprogramming of glucose metabolism is a cardinal feature of cell proliferation, we also assessed the effect of UPA or /and metformin on the expression of glucose-6-phosphate dehydrogenase (G6PD) and transketolase (TKT). It was observed that the uterine wall in the control group showed negative reactivity towards 3-PHGDH, G6PD and TKT in the endometrial glands and weak reactivity in the stromal cells. Uterine wall in the UPA treated group showed moderate reactivity towards 3-PHGDH but the metformin treated group and the UPA & metformin treated group showed weak reactivity in both the endometrial glands and stromal cells. In addition, uterine wall in the UPA treated group, the metformin treated group and the UPA & metformin treated group showed moderate, weak and mild reactivity respectively towards G6PD in both the endometrial glands and stromal cells. Uterine wall in the UPA treated group, the metformin treated group and the UPA & metformin treated group showed moderate, negative and weak reactivity respectively towards TKT in the endometrial glands and it showed marked, moderate and negative reactivity towards TKT in the UPA treated group, the metformin treated group and the UPA & metformin treated group respectively in stromal cells (Fig. 6).

Collectively, the immunoreactivity of 3-PHGDH in endometria from rats treated with UPA was significantly increased by one third- fold and 20-fold as compared with the control group and the group treated with metformin alone (0.62 ± 0.18 vs. 0.48 ± 0.12 and 0.02 ± 0.005; respectively) (*P* < 0.05). On the other hand, the immunoreactivity of 3-PGDH significantly decreased by half-fold in the group treated with metformin and UPA as compared to the group treated with UPA alone (0.24 ± 0.9 vs. 0.62 ± 0.18; respectively) (*P* < 0.05) (Fig. 6A–E). Our data also revealed that the immunoreactivity of G6PD was significantly higher by 1.5-fold in response to UPA as compared with both the control group and the group treated with metformin alone (0.7 ± 0.17 vs. 0.27 ± 0.08 and 0.25 ± 0.06; respectively, *P* < 0.05). The G6PD immunoreactivity was significantly reduced by half- fold after adding metformin to UPA when compared to UPA treated group (0.7 ± 0.17 vs. 0.37 ± 0.13; respectively, *P* < 0.05) (Fig. 6F–J). In addition, the immunoreactivity of TKT was significantly higher by half-fold and twofold in response to UPA as compared with the control group and the group treated with metformin alone (0.6 ± 0.05 vs. 0.4 ± 0.03 ± , 0.2 ± 0.06; respectively, *P* < 0.005). Addition of metformin to UPA did not show any effect on the expression of TKT when compared to the UPA treated group (Fig. 6K–O).

**Figure 7:**
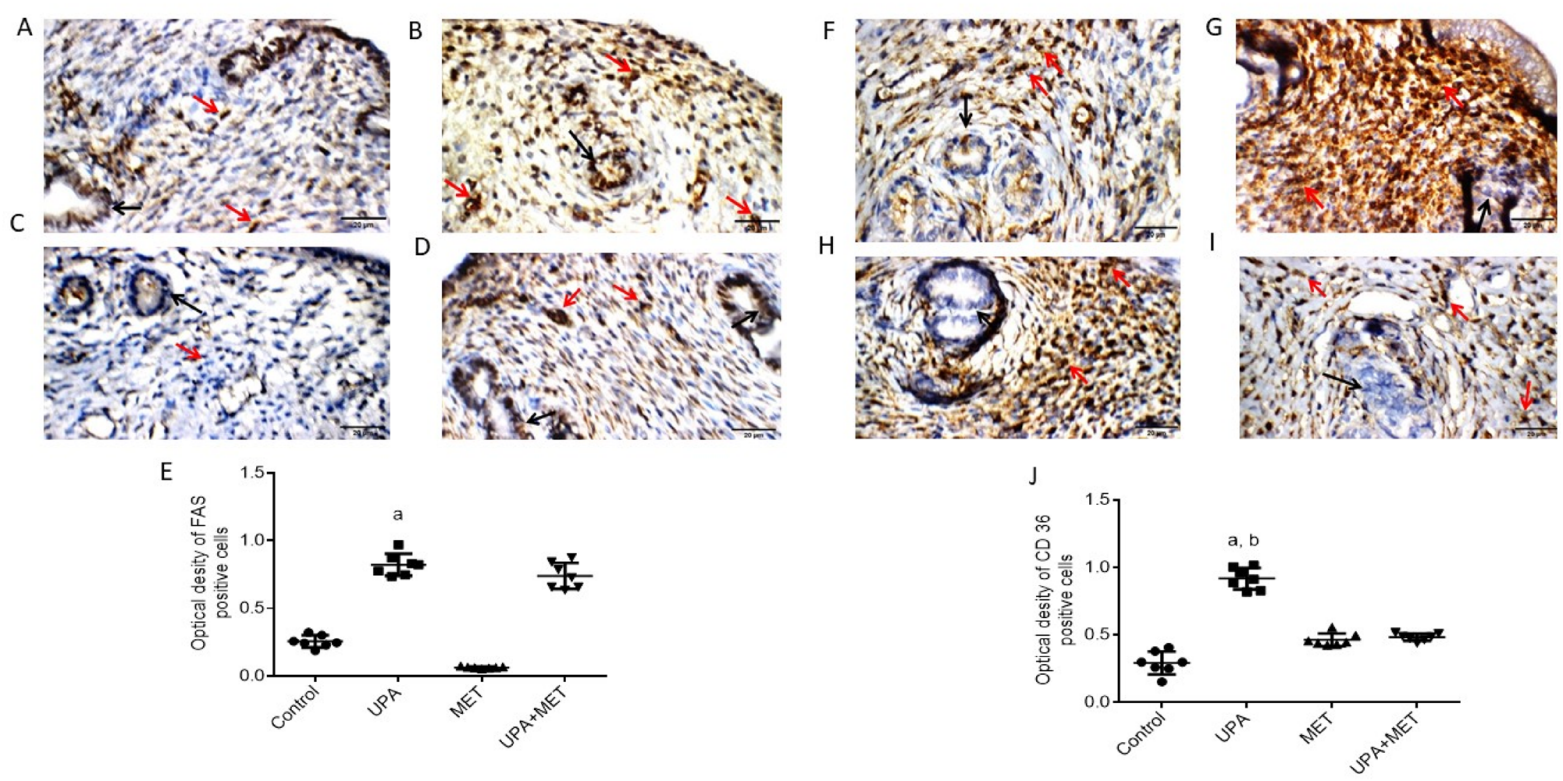
Effect of ulipristal and/or metformin on the expression of proteins involved in lipid metabolism in rats’ uteri. (**A**–**D**) Photomicrographs of uteri shows expression of FAS. (**A**) Uterine wall in the control group. (**B**) Uterine wall in ulipristal treated group. (**C**) Uterine wall in metformin treated group. (**D**) Uterine wall in the ulipristal and metformin treated group. × 400 magnification (scale bar = 20 μm). (**E**) Expression quantification of the optical density reactivity of positive cells of FAS by the ImageJ analysis system. (**F**–**I**) Photomicrographs of uteri shows expression of CD 36. (**F**) Uterine wall in the control group. (**G**) Uterine wall in ulipristal treated group. (H) Uterine wall in metformin treated group. (**I**) Uterine wall in the ulipristal and metformin treated group. × 400 magnification (scale bar = 20 μm). (**J**) Expression quantification of the optical density reactivity of positive cells of CD 36 by the ImageJ analysis system. Data figures (**E**) and (**J**) is represented by mean ± SD (n = 7). a or b, Statistically significant from the control or ulipristal and metformin treated group respectively at *P* < 0.05 using one-way ANOVA followed by Tukey as a post hoc test. *UPA* ulipristal, *MET* metformin, *UPA + MET* co-treatment with ulipristal and metformin.

Similarly, we examined the protein expression for both fatty acid synthase (FAS) and CD36, a scavenger receptor that is responsible for the uptake of long chain fatty acids. The uterine wall in the control group, the UPA treated group, the metformin treated group and the UPA & metformin treated group showed mild, marked, mild and weak reactivity respectively towards FAS in the endometrial glands. Also, it showed moderate reactivity in both the control and the UPA & metformin treated group towards FAS in the stromal cells while it showed marked and mild in the UPA treated group and the metformin group. Besides that, uterine wall in the control and metformin treated group showed weak and mild reactivity respectively towards CD36 in the endometrial glands and in the stromal cells. Although, it showed marked and moderate reactivity towards CD36 in the UPA treated group and the UPA & metformin treated in both the endometrial glands and in the stromal cells (Fig. 7).

Our data demonstrated that the expression of FAS was significantly higher by threefold and 12-fold in response to UPA treatment as compared with the control group and the group treated with metformin alone (0.8 ± 0.24 vs. 0.2 ± 0.07 and 0.06 ± 0.02; respectively, *P* < 0.01). The expression of FAS insignificantly decreased by 7% in the group treated with both UPA and metformin as compared to the UPA treated group (0.76 ± 0.3 vs. 0.8 ± 0.24) (Fig. 7A–E). In addition, the immunoreactivity of CD36 was significantly higher by twofold and onefold, respectively, in the uteri from the group treated with UPA as compared with the control group and the group treated with metformin alone (0.9 ± 0.25 vs. 0.3 ± 0.08 ± , 0.4 ± 0.16; respectively, *P* < 0.05). Addition of metformin to UPA significantly reduced immunoreactivity of CD36 by one half-fold when compared to the UPA treated group (0.9 ± 0.25 vs. 0.4 ± 0.14; respectively, *P* < 0.05) (Fig. 7F–J).

## Discussion

This study reported that the treatment of rats with Ulipristal acetate (UPA) for two consecutive months is associated with the development of some histological changes in rats’ endometrium consistent with progesterone receptor modulator-associated endometrial changes (PAECs). These UPA- induced PAECs were associated with altered expression of genes involved in cell proliferation, apoptosis as well as glucose and lipid metabolizing genes in endometrial tissues. More significantly, co-treatment with metformin mitigated UPA-induced PAECs in rats.

UPA is a selective progesterone receptor modulator (SPRM) which is proposed for the treatment of several gynecological conditions including uterine fibroids and endometriosis^4^. Also, UPA is taken as an emergency contraceptive within 120 h after contraceptive failure or unprotected sexual intercourse^32^. The regimen of daily doses of 5 mg or 10 mg UPA for several weeks has been studied for treatment of uterine fibroids and appears to be safe^6^. It is currently undergoing trials in the U.S. to evaluate its use as a daily contraceptive.

UPA exerts strong antagonistic and partial agonistic effects at PR in progesterone-responsive tissues^33^ and its use is associated with non-physiological reversible histopathological endometrial pathologic changes, collectively known as pathologic progesterone receptor modulator-associated endometrial changes (PAECs) which represent a serious concern among gynecologists^8^. In this experimental study, we attempted to illustrate the molecular mechanism by which UPA induces endometrial PAECs and the potential protective effect of metformin in alleviating such unwarranted effects. Thus, we investigated the effect of treatment with UPA and/or metformin on the expression of genes involved in apoptosis, cell proliferation and cellular metabolism in rats. However, it would be interesting to reproduce these findings in animal models of uterine fibroid such as EKER rats or mice model of uterine fibroids.

To study the effect of UPA and metformin on proliferation in rats’ uteri, we measured the expression level of cyclin D1 and PCNA. Metformin decreased the expression of cyclin D1 and PCNA which are associated with cell cycle and tumor induction, respectively. In addition, the present study found that administration of metformin along with UPA is effective in resolving benign endometrial proliferative lesions. Our results were in line with previous preclinical studies that examined the anti-proliferative role of metformin in treatment of endometrial cell growth^34–39^. On the other hand, a previous study demonstrated that UPA did not increase the proliferation of human endometrium^40^, which is in contrast to our finding that UPA increased proliferation in rats’ endometrium. This apparent difference in our finding and the finding in human endometrium might be explained by the qualitative and quantitative physiological differences between rats and human endometrium. For instance, a recent study demonstrated that primary human and rat endometrial cells respond differently towards hormones and nuclear hormone receptor ligands and this should be considered in human risk assessment based on rodent studies^41^.

Moreover, the results showed that UPA decreases apoptosis in rats’ endometrial tissues and metformin reverses this effect by downregulating the expression of antiapoptotic Bcl-2 and upregulating the pro-apoptotic Bax expression. These findings were in line with a previous study that demonstrated that metformin suppressed cell death by modulating the Bax pathway^42^.

Our results suggested that UPA-induced endometrial changes in rats are attributed, at least in part, to its effects on the cellular metabolism. In fact, progesterone/progesterone receptors play important roles in regulating cellular metabolism, including lipid metabolism, steroid biosynthesis and metabolism, glucose metabolism, regulation of cell cycle and proliferation, cell migration and invasion^43^. Thus, modulation of progesterone receptors by UPA can cause dysregulation of the cellular metabolism and increase risk of PAECs. These findings can be explained in light of the well-established fact that sex hormones and their cognate receptors reprogram the metabolism in endometrial cells. Moreover, progesterone/PR regulates the expression of genes involved glycolysis and lipid metabolism in endometrium^17,44,45^. The regulatory effects of progesterone/PR on the expression of glycolytic and lipid-metabolizing genes could be direct or indirect. Progesterone/PR could regulate the expression of genes involved in cellular metabolism indirectly via activation of other transcription factors such as Hif1α and c-Myc, HAND2, FOXO1, FOXM1, or through activation of Pi3k-Akt signaling pathway^46,47^.

Furthermore, previous finding showed that treatment with UPA increases the expression of ERα in endometrial tissues to the level comparable to a level in the proliferative phase of the endometrium^48^. Thus, it is likely that the effect of UPA on ERα expression may also contribute to the observed changes in the glycolytic genes and lipid-metabolizing genes expressions. In fact, the expression of many genes involved in glucose and lipid metabolism are regulated by sex steroid hormones and their cognate receptors^17,49,50^.

The biguanide metformin is used in the treatment of type 2 diabetes and concrete evidence indicates that metformin may have beneficial therapeutic effects in endometrial tissues. Indeed, our results demonstrated that metformin prevents UPA-induced endometrial pathological changes. It has been reported that the beneficial therapeutic effects of metformin on the endometrial tissues are related to its effects within the metabolic milieu. Metformin can inhibit cell growth, proliferation and promote the apoptosis. That occurs by inhibiting glycolysis energy metabolism through targeting several signaling pathways including PI3K/Akt/mTOR^51^ and AMPK activation^52^. Similarly, emerging evidence demonstrated that metformin inhibits cell proliferation and decreases glycolytic flux through the HIF-1α/PFKFB3/PFK1 pathway^53^. It has been reported that metformin inhibits the expression of CD36 expression^54^, and the expression of FAS and HK-2 by targeting c-Myc in an AMPK-dependent manner^55^.

Our study also clearly demonstrated that treatment with UPA resulted in a significant increase in ERα expression in the endometrial tissues. This finding is consistent with the previous finding that, ERα expression in endometrial tissues in patients treated with UPA is similar to the proliferative phase in the endometrium, although with a decrease in cell proliferation^40,48^. UPA-induced expression of ER α might play a role in UPA-induced PAECs. It has been reported that endometrial proliferation/hyperplasia usually develops in the presence of continuous estrogen stimulation unopposed by progesterone^56^.

Our study also revealed that metformin abrogated UPA-induced ERα expression in endometrial tissues. This observation is in harmony with an earlier study demonstrating that metformin significantly decreased ER α in endometrial cells^57^. The effect of metformin on the ER α expression might explain, in part, its beneficial therapeutic effects against UPA-induced endometrial changes which is associated with increased ER α expression.

Collectively, these findings have suggested the beneficial therapeutic effect of metformin against UPA-induced PAECs in rats. However, the clinical relevance of these findings is still uncertain and preclinical and clinical studies are warranted. In conclusion, UPA induced PAECs are associated with altered expression of genes involved in cell cycle, apoptosis and ERα. Most importantly, UPA alters the expression of genes involved in glucose and lipid metabolism, which significantly contribute to the development of PAECs. Metformin abrogates UPA-induced endometrial PAECs through modulating the expression of glycolytic and lipid-metabolizing genes. Thus, metformin might represent a clinically visible option to mitigate the unwanted effects of UPA in endometrial tissues.

A limitation of the study is that extrapolation of our finding in rats to women requires careful attention to potentially important differences between the physiological and pharmacological response of the two species to the treatment with UPA. Another limitation is that it would be optimal to study the effect of metformin on UPA-induced endometrial changes in animal model with uterine fibroids.

## Materials and methods

### Chemicals and reagents

UPA was purchased from Henan FoTei Biological Technology Co., Ltd. (HeNan, China) with purity more than 98%. The following antibodies were obtained from Santa Cruz Biotechnology (Santa Cruz, CA, USA): mouse anti-rat Transketolase (Cat# sc-390179), mouse anti-rat Bax (Cat# sc-20067), mouse anti-rat Bcl-2 (Cat# sc-7382), mouse anti-rat CD36 (Cat# sc-7309), mouse anti-rat Fatty Acid Synthase (Cat# sc-55580), mouse anti-rat G6PD (Cat# sc-373886), mouse anti-rat PCNA (Cat# sc-25280), mouse anti-rat cyclin D1 (Cat# sc-246), mouse anti-rat 3-PGDH (Cat# sc- 390610), mouse anti-rat ERα (Cat# sc-8005) and mouse anti-rat PR (Cat# sc-398898). Other chemicals were of the highest analytical grade available commercially.

### Animals and treatment protocol

All animal procedures were ethically approved by the Research Ethics Committee, Faculty of Pharmacy, the British University in Egypt, Cairo, Egypt. In addition, all procedures were performed in accordance with US government guidelines for utilization and care of vertebrate animals used in testing, research, and training and the Animal Research: Reporting In Vivo Experiments (ARRIVE) guidelines. Twenty-eight female non-pregnant, nulligravid Wistar rats (8-week-old female weighing 180–200 g) were obtained from the animal house in Faculty of Pharmacy, the British University in Egypt. Rats were kept on a half-day light/dark cycle in air conditioning. Animals’ acclimatization was extended for 1 week before the study.

### Experimental design

After acclimatization, the animals were fed ad libitum. Rats were then randomized into four treatment groups (7 rats/each):

- The first group served as negative control and was treated with the vehicle used for UPA, (distilled water and ethyl alcohol; 4:1).
- The second group was treated with UPA (0.1 mg/kg/day, intragastric) for 8 weeks^58^.
- The third group was treated with metformin dissolved in distilled water and ethyl alcohol; 4:1 (50 mg/kg/day, intragastric) for 8 weeks^59^.
- The forth group was treated with UPA (0.1 mg/kg/day; intragastric) + metformin 50 mg/kg/day, intragastric) for 8 weeks.

Rats were monitored daily, and no evidence of toxicity was recorded under the current treatment protocol.

### Isolation of endometrial tissues

The rats were sacrificed by neck fracture, the uterus from each animal was completely separated and weighed at the end of the experiment. Uterine horns were split lengthwise and total endometrial tissues were scrapped with a fine scalpel. Part of the endometrial tissues were kept at − 80 °C. The other part of the uterus sections was collected in 10% buffered formalin, fixed overnight, and embedded in paraffin blocks for histological and immunohistochemical examinations. Paraffin blocks were cut at 4 mm thickness and stained with hematoxylin and eosin (H&E) per standard protocol^60^.

### Determination of uterus weight and uterus index

Uteri were harvested and weighed instantly then uterus index was calculated as the ratio of the uterus weight to the total body weight.

### Histopathological examination

The H & E stained slides were visualized and the images were captured digitally using light microscopy (Nikon Eclipse TE2000-U, NIKON, Japan). An experienced pathologist who was blinded to the experimental design performed morphometric analysis on mid-horn uterine cross sections.

### Determination of Uterine gland cross-sections and endometrium thickness

The images of the H&E stained slides were used to determine uterine gland cross-sections and endometrium thickness by ImageJ software (ImageJ, 1.46a, NIH, USA). Briefly, microscopic fields (3 for thickness, 6 for area) per rat were randomly selected. Uterine gland cross-sections per uterine horn were calculated by determining the number of cross-sections in 1 µm and then multiplying by uterine horn length. All measurements were made blinded to treatment group.

### Immunohistochemistry (IHC)

To examine the effect of UPA and/or metformin in endometrial tissues, we used immunohistochemical staining (IHC) of several proteins that are involved in, apoptosis (Bax and Bcl-2), cell proliferation (PCNA and Cyclin D1), glucose metabolism (3-PGDH, G6PD and Transketolase), and fat metabolism (Fatty acid synthase and CD36). In addition, the expression of sex hormones receptors (ERα and PR) were also assessed.

For the immunohistochemical staining, the tissue sections were incubated with 1% BSA in PBS at room temperature for 1 h., follow by incubating with the intended primary antibodies in 4 °C for 15 h. After incubation with the primary antibody, slides were flushed by TBS. Then the slides were incubated with the biotinylated secondary antibody using Cell and Tissue Staining Kit (R&D Systems, MN, USA). Negative controls were obtained by incubating sections with phosphate-buffered saline (PBS) instead of primary antibodies (Supplemental Fig. 1). Image analysis was performed by ImageJ analysis software (ImageJ, 1.46a, NIH, USA). Quantification of immunoscore of all the images was blinded and was done according to the standard recommended protocol^61^. The images were converted to grayscale, and the black level was set to reduce the level of overstaining on a stained image. We tested several threshold values to distinguish the areas with an immunostaied signal from the background. The threshold with the best outcomes was applied to all images, and the area of signal was measured. Twenty microscopic fields were examined for each slide, and the average positive area was determined. Validation of ImageJ analysis was performed by an experienced pathologist who was blinded to the experimental design.

### Quantitative real time RT-PCR analysis

Real-time PCR experiments were carried out In agreement with the MIQE guidelines for qPCR^62^. Total RNA was extracted by GeneJET RNA Purification Kit (Thermo Fisher Scientific, Massachusetts, USA) according to manufacturer’s protocol. One microgram of RNA was reverse transcribed using High-Capacity cDNA Reverse Transcription Kit (Thermo Fisher Scientific, Massachusetts, USA) with random primer according to the manufacturer’s instruction. From each treatment group, we used cDNA from all the rats to perform quantitative real time RT-PCR analysis. One-tenth of the resulting cDNA was used as a template for real-time PCR amplification using Maxima SYBR Green qPCR master mix (Thermo Fisher Scientific, Massachusetts, USA). For each sample, the quantitative Real-time PCR was performed in triplicates in StepOne Real-Time PCR System (applied bio systems) using the SYBR Green method. Specific primers for Bax, Bcl-2, Cyclin d1, and PCNA. GAPDH was amplified and used as endogenous control to normalize for gene expression; it is confirmed that GAPDH does not change with experimental conditions. A no template control (NTC) was used as negative control using diethylpyrocarbonate (DEPC) treated water. The primers used for the amplification of the indicated genes were designed using the IDT Primer Quest Primer Design Tool and are listed in Table 1. Data were analyzed and plotted using the change in cycle threshold (ΔCt) method for the calculation of relative changes in gene expression. In addition, fold change in gene expression were determined using the 2^−ΔΔ*CT*^ method^63^.

**Table 1:** Primers used for real-time PCR assays.

Name of the primer	5′-3'
Bax-F	GGCGAATTGGAGATGAACTG
Bax-R	CCCCAGTTGAAGTTGCCAT
Bcl-2-F	GATGACTGAGTACCTGAACCG
Bcl-2-R	CAGAGACAGCCAGGAGAAATC
Cyclin D1-F	CTACACTGACAACTCTATCCGC
Cyclin D1-R	TCTGGCATTTTGGAGAGGAAG
GAPDH-F	CAGTGCCAGCCTCGTCTCAT
GAPDH-R	CAAGAGAAGGCAGCCCTGGT
PCNA-F	TGGTGATCTCCTGTGCAAAG
PCNA-R	CAAAAGTTAGCTGAACTGGCTC

Thermal cycle parameters were: 1. Cycle at 95 °C for 15 min followed by 35 cycles at 95 °C for 10 s, annealing at 61 °C for 20 s and at 72 °C for 20 s.

### Statistical analysis

Results are shown as mean ± SD. One-way ANOVA followed by Tukey as a post hoc test was used to conduct multiple comparisons to assess the statistical significance between different groups. Differences between means were considered significant at *P* $\mathrm{value }\le 0.05$. GraphPad Prism 6 was used for carrying out statistical analysis. Data from RT-PCR were analyzed and plotted using the change in dCT values (ΔCt).

## Supplementary Information

Supplementary Figure 1.

## References

[1] Gainer EE, Ulmann A (2003). Pharmacologic properties of CDB(VA)-2914. Steroids.

[2] Attardi BJ, Burgenson J, Hild SA, Reel JR (2004). In vitro antiprogestational/antiglucocorticoid activity and progestin and glucocorticoid receptor binding of the putative metabolites and synthetic derivatives of CDB-2914, CDB-4124, and mifepristone. J. Steroid Biochem. Mol. Biol..

[3] Williams AR, Bergeron C, Barlow DH, Ferenczy A (2012). Endometrial morphology after treatment of uterine fibroids with the selective progesterone receptor modulator, ulipristal acetate. Int. J. Gynecol. Pathol..

[4] Chabbert-Buffet N, Meduri G, Bouchard P, Spitz IM (2005). Selective progesterone receptor modulators and progesterone antagonists: mechanisms of action and clinical applications. Hum. Reprod. Update.

[5] Spitz IM, Croxatto HB, Robbins A (1996). Antiprogestins: mechanism of action and contraceptive potential. Annu. Rev. Pharmacol. Toxicol..

[6] Donnez J (2012). Ulipristal acetate versus placebo for fibroid treatment before surgery. N. Engl. J. Med..

[7] Donnez J (2012). Ulipristal acetate versus leuprolide acetate for uterine fibroids. N. Engl. J. Med..

[8] Mutter GL (2008). The spectrum of endometrial pathology induced by progesterone receptor modulators. Mod. Pathol..

[9] Chandra V, Kim JJ (2016). Therapeutic options for management of endometrial hyperplasia. J. Gynecol. Oncol..

[10] Horn LC, Schnurrbusch U, Bilek K, Hentschel B, Einenkel J (2004). Risk of progression in complex and atypical endometrial hyperplasia: clinicopathologic analysis in cases with and without progestogen treatment. Int. J. Gynecol. Cancer.

[11] Donnez J (2014). Long-term treatment of uterine fibroids with ulipristal acetate. Fertil. Steril..

[12] Wagenfeld A, Saunders PT, Whitaker L, Critchley HO (2016). Selective progesterone receptor modulators (SPRMs): progesterone receptor action, mode of action on the endometrium and treatment options in gynecological therapies. Expert Opin. Ther. Targets.

[13] Cruz MD (2017). Metabolic reprogramming of the premalignant colonic mucosa is an early event in carcinogenesis. Oncotarget.

[14] Fu Y (2017). The reverse Warburg effect is likely to be an Achilles' heel of cancer that can be exploited for cancer therapy. Oncotarget.

[15] Al-Zoughbi W (2014). Tumor macroenvironment and metabolism. Semin. Oncol..

[16] Deblois G, Giguère V (2013). Oestrogen-related receptors in breast cancer: control of cellular metabolism and beyond. Nat. Rev. Cancer.

[17] Salama SA (2014). Estradiol-17β upregulates pyruvate kinase M2 expression to coactivate estrogen receptor-α and to integrate metabolic reprogramming with the mitogenic response in endometrial cells. J. Clin. Endocrinol. Metab..

[18] Ko EM (2014). Metformin is associated with improved survival in endometrial cancer. Gynecol. Oncol..

[19] Lv Z, Guo Y (2020). Metformin and its benefits for various diseases. Front. Endocrinol..

[20] Griss T (2015). Metformin antagonizes cancer cell proliferation by suppressing mitochondrial-dependent biosynthesis. PLoS Biol..

[21] Kato H (2015). Metformin inhibits the proliferation of human prostate cancer PC-3 cells via the downregulation of insulin-like growth factor 1 receptor. Biochem. Biophys. Res. Commun..

[22] Shi WY (2012). Therapeutic metformin/AMPK activation blocked lymphoma cell growth via inhibition of mTOR pathway and induction of autophagy. Cell Death Dis..

[23] Shen ZQ, Zhu HT, Lin JF (2008). Reverse of progestin-resistant atypical endometrial hyperplasia by metformin and oral contraceptives. Obstet. Gynecol..

[24] Javanmanesh F, Kashanian M, Rahimi M, Sheikhansari N (2016). A comparison between the effects of metformin and N-acetyl cysteine (NAC) on some metabolic and endocrine characteristics of women with polycystic ovary syndrome. Gynecol. Endocrinol..

[25] Sivalingam VN (2016). Measuring the biological effect of presurgical metformin treatment in endometrial cancer. Br. J. Cancer.

[26] Mitsuhashi A (2016). Phase II study of medroxyprogesterone acetate plus metformin as a fertility-sparing treatment for atypical endometrial hyperplasia and endometrial cancer. Ann. Oncol..

[27] Papanas N, Maltezos E, Mikhailidis DP (2010). Metformin and cancer: licence to heal?. Expert Opin. Investig. Drugs.

[28] Calle EE, Rodriguez C, Walker-Thurmond K, Thun MJ (2003). Overweight, obesity, and mortality from cancer in a prospectively studied cohort of U.S. adults. N. Engl. J. Med..

[29] Giudice LC (2006). Endometrium in PCOS: implantation and predisposition to endocrine CA. Best Pract. Res. Clin. Endocrinol. Metab..

[30] Garmes HM, Tambascia MA, Zantut-Wittmann DE (2005). Endocrine-metabolic effects of the treatment with pioglitazone in obese patients with polycystic ovary syndrome. Gynecol. Endocrinol..

[31] Zhang B (2017). PHGDH defines a metabolic subtype in lung adenocarcinomas with poor prognosis. Cell Rep..

[32] McKeage K, Croxtall JD (2011). Ulipristal acetate: a review of its use in emergency contraception. Drugs.

[33] Chwalisz K (2005). Selective progesterone receptor modulator development and use in the treatment of leiomyomata and endometriosis. Endocr. Rev..

[34] Zhou G (2001). Role of AMP-activated protein kinase in mechanism of metformin action. J. Clin. Investig..

[35] Fryer LG, Parbu-Patel A, Carling D (2002). The anti-diabetic drugs rosiglitazone and metformin stimulate AMP-activated protein kinase through distinct signaling pathways. J. Biol. Chem..

[36] Hardie DG (2003). Minireview: the AMP-activated protein kinase cascade: the key sensor of cellular energy status. Endocrinology.

[37] Zakikhani M, Dowling R, Fantus IG, Sonenberg N, Pollak M (2006). Metformin is an AMP kinase-dependent growth inhibitor for breast cancer cells. Can. Res..

[38] Huang X (2008). Important role of the LKB1-AMPK pathway in suppressing tumorigenesis in PTEN-deficient mice. Biochem. J..

[39] Tabrizi AD, Melli MS, Foroughi M, Ghojazadeh M, Bidadi S (2014). Antiproliferative effect of metformin on the endometrium—a clinical trial. Asian Pac. J. Cancer Prev. APJCP.

[40] Whitaker LH (2017). Selective progesterone receptor modulator (SPRM) ulipristal acetate (UPA) and its effects on the human endometrium. Hum. Reprod. (Oxf., Engl.).

[41] van den Brand AD, Rubinstein E, de Jong PC, van den Berg M, van Duursen M (2020). Assessing anti-estrogenic effects of AHR ligands in primary human and rat endometrial epithelial cells. Reprod. Toxicol. (Elmsford, N.Y.).

[42] Sahin E (2018). Induction of apoptosis by metformin and progesterone in estrogen-induced endometrial hyperplasia in rats: involvement of the bcl-2 family proteins. Gynecol. Endocrinol..

[43] Sabbir MG (2019). Progesterone induced Warburg effect in HEK293 cells is associated with post-translational modifications and proteasomal degradation of progesterone receptor membrane component 1. J. Steroid Biochem. Mol. Biol..

[44] Barros RP, Gustafsson J (2011). Estrogen receptors and the metabolic network. Cell Metab..

[45] Boonyaratanakornkit V, Pateetin P (2015). The role of ovarian sex steroids in metabolic homeostasis, obesity, and postmenopausal breast cancer: molecular mechanisms and therapeutic implications. Biomed. Res. Int..

[46] Whitaker LHR (2017). Selective progesterone receptor modulator (SPRM) ulipristal acetate (UPA) and its effects on the human endometrium. Hum. Reprod..

[47] Zuo RJ (2015). Warburg-like glycolysis and lactate shuttle in mouse decidua during early pregnancy. J. Biol. Chem..

[48] Kolanska K (2019). Absence of predictable long-term molecular effect of ulipristal acetate (UPA) on the endometrium. Reprod. Biomed. Online.

[49] Fukata Y (2014). 17β-Estradiol regulates scavenger receptor class BI gene expression via protein kinase C in vascular endothelial cells. Endocrine.

[50] Yang X (2016). Activation of peroxisome proliferator-activated receptor γ (PPARγ) and CD36 protein expression: the dual pathophysiological roles of progesterone. J. Biol. Chem..

[51] Tang JC, An R, Jiang YQ, Yang J (2017). Effects and mechanisms of metformin on the proliferation of esophageal cancer cells in vitro and in vivo. Cancer Res. Treat..

[52] Li D (2011). Metformin as an antitumor agent in cancer prevention and treatment. J. Diabetes.

[53] Hu L (2019). Metformin attenuates hepatoma cell proliferation by decreasing glycolytic flux through the HIF-1α/PFKFB3/PFK1 pathway. Life Sci..

[54] Moon JS (2017). Metformin prevents glucotoxicity by alleviating oxidative and ER stress-induced CD36 expression in pancreatic beta cells. J. Diabetes Complic..

[55] Zhang C (2019). Metformin delays AKT/c-Met-driven hepatocarcinogenesis by regulating signaling pathways for de novo lipogenesis and ATP generation. Toxicol. Appl. Pharmacol..

[56] Epplein M (2008). Risk of complex and atypical endometrial hyperplasia in relation to anthropometric measures and reproductive history. Am. J. Epidemiol..

[57] Collins G, Mesiano S, DiFeo A (2019). Effects of metformin on cellular proliferation and steroid hormone receptors in patient-derived, low-grade endometrial cancer cell lines. Reprod. Sci. (Thousand Oaks, Calif.).

[58] Huniadi CA, Pop OL, Antal TA, Stamatian F (2013). The effects of ulipristal on Bax/Bcl-2, cytochrome c, Ki-67 and cyclooxygenase-2 expression in a rat model with surgically induced endometriosis. Eur. J. Obstet. Gynecol. Reprod. Biol..

[59] Thompson MD (2017). Lack of chemopreventive efficacy of metformin in rodent models of urinary bladder, head and neck, and colon/intestine cancer. Oncol. Lett..

[60] Cardiff RD, Miller CH, Munn RJ (2014). Manual hematoxylin and eosin staining of mouse tissue sections. Cold Spring Harb. Protoc..

[61] Schneider CA, Rasband WS, Eliceiri KW (2012). NIH Image to ImageJ: 25 years of image analysis. Nat. Methods.

[62] Bustin SA (2009). The MIQE guidelines: minimum information for publication of quantitative real-time PCR experiments. Clin. Chem..

[63] Livak KJ, Schmittgen TD (2001). Analysis of relative gene expression data using real-time quantitative PCR and the 2(-Delta Delta C(T)) method. Methods (San Diego, Calif.).

