# Author Correction: Glucose and fatty acid metabolism involved in the protective effect of metformin against ulipristal-induced endometrial changes in rats

**DOI:** 10.1038/s41598-021-97931-y

**Published:** 2021-09-08

**Authors:** Marwa S. Hamza, Eman Ramadan, Salama A. Salama

**Affiliations:** 1 Clinical Pharmacy Practice Department, Faculty of Pharmacy, The British University in Egypt, El-Sherouk City, P.O. Box 43, Cairo, 11837 Egypt; 2 The Center for Drug Research and Development (CDRD), Faculty of Pharmacy, The British University in Egypt, El-Sherouk City, P.O. Box 43, Cairo, 11837 Egypt; 3 Pharmacology and Biochemistry Department, Faculty of Pharmacy, The British University in Egypt, El-Sherouk City, P.O. Box 43, Cairo, 11837 Egypt; 4 Pharmacology and Toxicology Department, Faculty of Pharmacy, Al-Azhar University Nasr City, Cairo, 11751 Egypt

Correction to: *Scientific Reports* 10.1038/s41598-021-88346-w, published online 23 April 2021

In the original version of the Article, Figure 4 was a duplication of Figure 3.

The original Figure 4 and accompanying legend appear below.

**Figure 4:**
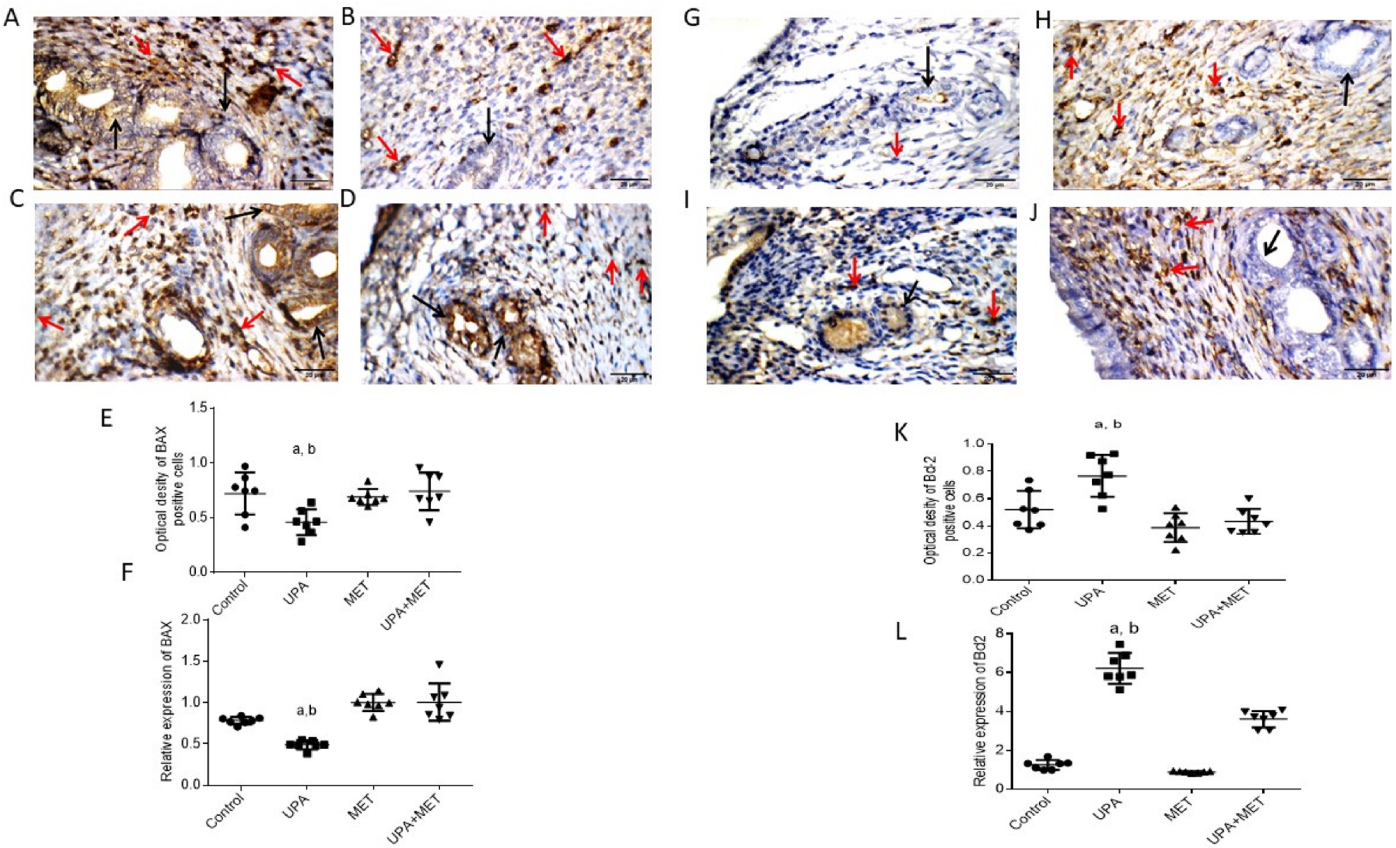
Effect of metformin or/and ulipristal on the expression of the proliferation markers of rats’ uteri. (**A**–**D**) photomicrographs of uteri shows expression of PCNA (**A**) Uterine wall in the control group. (**B**) Uterine wall in ulipristal treated group. (**C**) Uterine wall in metformin treated group. (**D**) Uterine wall in the ulipristal and metformin treated group. × 400 magnification (scale bar = 20 μm). (**E**) Expression quantification of the optical density reactivity of positive cells of PCNA by the ImageJ analysis system. (**F**) Quantitative RT-PCR analysis of PCNA mRNA expression. (**G**–**J**) Photomicrographs of uteri shows expression of Cyclin-D. (**G**) Uterine wall in the control group. (**H**) Uterine wall in ulipristal treated group. (I) Uterine wall in metformin treated group. (**J**) Uterine wall in the ulipristal and metformin treated group. × 400 magnification (scale bar = 20 μm). (**K**) Expression quantification of the optical density reactivity of positive cells by the ImageJ analysis system. (**L**) Quantitative RT-PCR analysis of Cyclin D1 mRNA expression, Black arrow refers to reactivity in endometrial glands, red arrows refers to reactivity in stromal cells. Data in figures (**E**) and (**K**) is represented by mean ± SD (n = 7). All value of RT-PCR is expressed as the change in cycle threshold (ΔCt). Each dot represents mean of each group (n = 7, triplicate for each rat). Negative control (DEPC-treated water) showed no detectable fluorescent signals. a or b, Statistically significant from the control or ulipristal and metformin treated group respectively at *P* < 0.05 using one-way ANOVA followed by Tukey as a post hoc test. *UPA* ulipristal, *MET* metformin, *UPA* + *MET* co-treatment with ulipristal and metformin.

The original Article has been corrected.

