# Supplementary material for: Glucose and fatty acid metabolism involved in the protective effect of metformin against ulipristal-induced endometrial changes in rats
Source: Sci Rep. 2021 Apr 23;11:8863. doi: 10.1038/s41598-021-88346-w (PMC8065147; doi:10.1038/s41598-021-88346-w)
Supplement: Supplementary file 1 — Supplementary Figure 1. [file 41598_2021_88346_MOESM1_ESM.docx]

**Supplemental Figure Legends:**

**Supplemental Figure 1. Immunohistochemical staining for the expression of all the markers was optimized using sections as negative controls. (A) Omission of primary anti-Bax antibody. (B) Omission of primary anti-Bcl-2 antibody. (C) Omission of primary anti- PCNA antibody. (D) Omission of primary anti- Cyclin-D antibody. (E) Omission of primary anti- ERα antibody. (F) Omission of primary anti- PR antibody. (G) Omission of primary anti- 3-PHGDH antibody. (H) Omission of primary anti- G6PD antibody. (I) Omission of primary anti- TKT antibody. (J) Omission of primary anti- FAS antibody. (K) Omission of primary anti- CD36 antibody. x400 magnification (scale bar = 20 μm).**

Black arrow refers to negative reactivity in endometrial glands and red arrows refers to negative reactivity in the stromal cells.

**
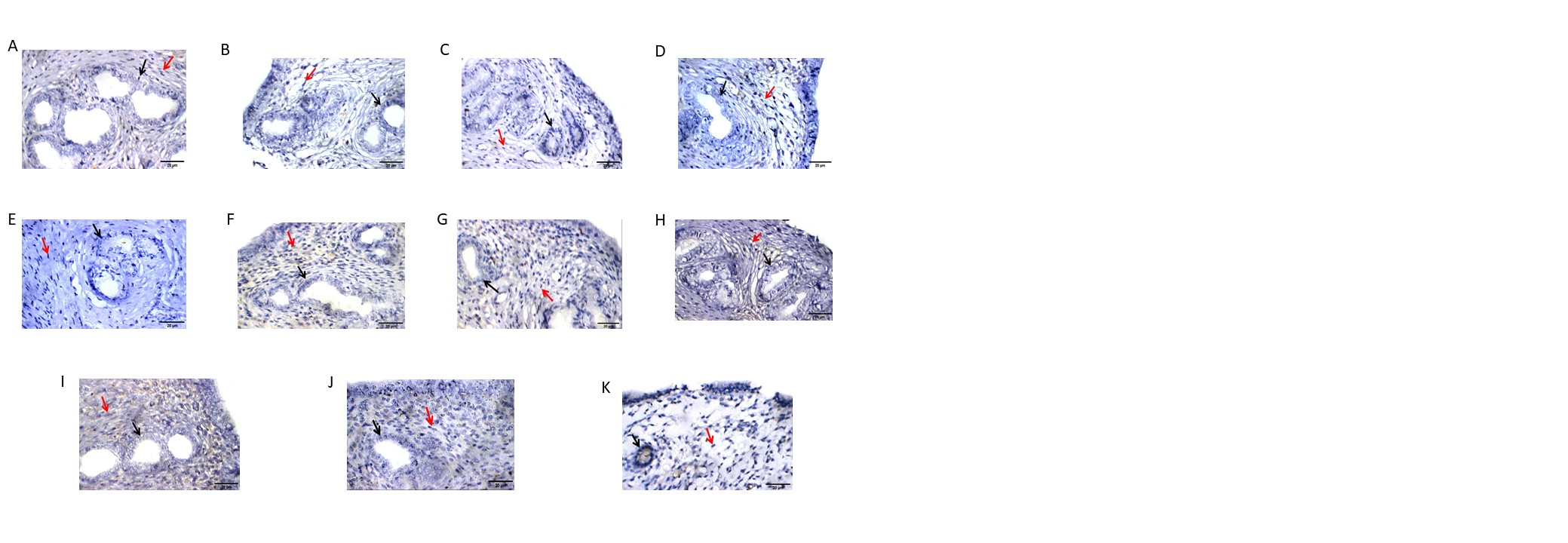
**
